# P-1897. Employing Interactive Case-Based Continuing Education to Improve HIV Decision-Making

**DOI:** 10.1093/ofid/ofaf695.2066

**Published:** 2026-01-11

**Authors:** Bharati Hegde, Jennifer Frederick, Katie Robinson, Stephanie M Johnson

**Affiliations:** Vindico Medical Education, Thorofare, New Jersey; Vindico Medical Education, Thorofare, New Jersey; Vindico Medical Education, Thorofare, New Jersey; Vindico Medical Education, Thorofare, New Jersey

## Abstract

**Background:**

As HIV management grows increasingly complex, infectious disease (ID) clinicians continue to face challenges and must make nuanced decisions to improve patient outcomes.

**Figure:**
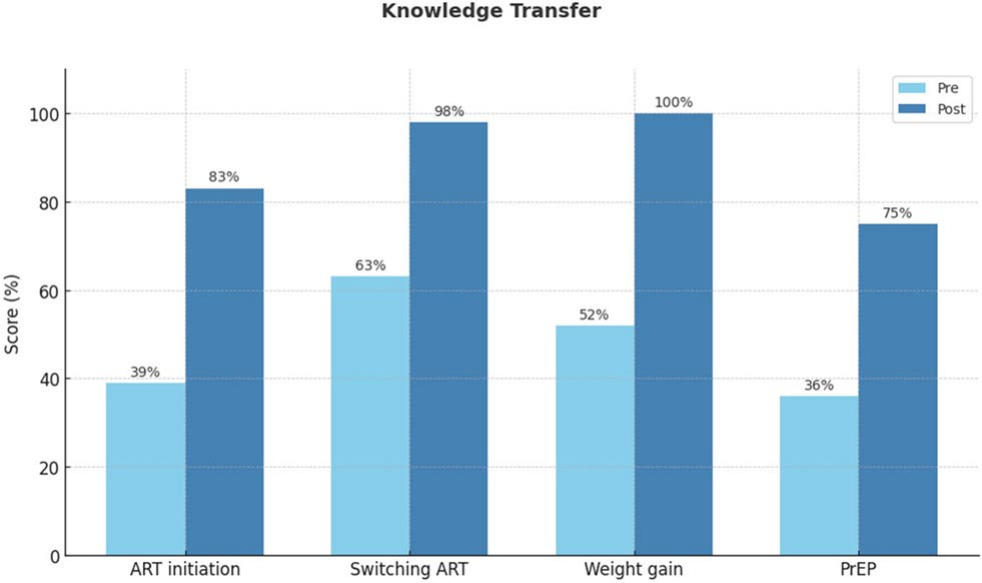
Knowledge Gains Across Topics

**Methods:**

Vindico Medical Education provided a live continuing education (CE) symposium featuring interactive cases at IDWeek 2024. Through real-time polling and expert-guided discussions, clinicians made decisions in realistic clinical scenarios related to antiretroviral therapy (ART) initiation, switching ART, weight management, and use of pre-exposure prophylaxis (PrEP). Baseline and post-education knowledge and confidence were measured via pre- and post-test data.

**Results:**

A total of 206 clinicians involved in the management of patients with HIV attended the symposium. At baseline, 61% of participants did not identify the appropriate timing for initiating ART in a patient with advanced HIV, 37% were unfamiliar with outcomes associated with switching ART, and 64% did not recognize the US FDA/CDC-recommended screening for PrEP (Figure 1). Overall, knowledge increased 42%. Improvements in behaviors were also noted. For instance, while 48% of participants at baseline could not appropriately manage a patient with ART-associated weight gain, following discussion of a related case scenario, 60% recommended a GLP-1 receptor agonist, indicating alignment of knowledge with evidence-based decision-making. Similarly, at baseline, half of the participants noted that lack of awareness about PrEP was the biggest challenge in implementation; post-education, however, 99% plan to use strategies to improve the use of PrEP. Additionally, post-learning, there was a 44% increase in competence regarding when to initiate ART in a patient with a co-infection. At the end of the education, 83% were confident in making decisions related to switching ART.

**Conclusion:**

Staying current on the latest evidence-based care regarding HIV PrEP and treatment is a persistent challenge for clinicians. This CE activity promoted significant improvements in knowledge, confidence, and decision making among clinicians who treat patients with and at risk for HIV. Such interactive, case-based CE is an important tool that can be used to address practice gaps as the HIV landscape continues to evolve.

**Disclosures:**

All Authors: No reported disclosures

